# Nanoquartz in Late Permian C1 coal and the high incidence of female lung cancer in the Pearl River Origin area: a retrospective cohort study

**DOI:** 10.1186/1471-2458-8-398

**Published:** 2008-12-02

**Authors:** Linwei Tian, Shifeng Dai, Jianfang Wang, Yunchao Huang, Suzanne C Ho, Yiping Zhou, Donald Lucas, Catherine P Koshland

**Affiliations:** 1 School of Public Health, Chinese University of Hong Kong, Hong Kong SAR, PR China; 2 State Key Laboratory of Coal Resources & Safe Mining, Chinese University of Mining and Technology, Beijing 100083, PR China; 3 Department of Physics, Chinese University of Hong Kong, Hong Kong SAR, PR China; 4 Yunnan Province Tumor Hospital and The Third Affiliated Hospital of Kunming Medical University, Kunming 650106, PR China; 5 Yunnan Institute of Coal Geology Prospection, Kunming 650218, PR China; 6 Lawrence Berkeley National Laboratory, University of California, Berkeley, CA 94720, USA; 7 School of Public Health, University of California, Berkeley, CA 94720, USA; 8 Energy and Resources Group, University of California, Berkeley, CA 94720, USA

## Abstract

**Background:**

The Pearl River Origin area, Qujing District of Yunnan Province, has one of the highest female lung cancer mortality rates in China. Smoking was excluded as a cause of the lung cancer excess because almost all women were non-smokers. Crystalline silica embedded in the soot emissions from coal combustion was found to be associated with the lung cancer risk in a geographical correlation study. Lung cancer rates tend to be higher in places where the Late Permian C1 coal is produced. Therefore, we have hypothesized the two processes: C1 coal combustion --> nanoquartz in ambient air --> lung cancer excess in non-smoking women.

**Methods/Design:**

We propose to conduct a retrospective cohort study to test the hypothesis above. We will search historical records and compile an inventory of the coal mines in operation during 1930–2009. To estimate the study subjects' retrospective exposure, we will reconstruct the historical exposure scenario by burning the coal samples, collected from operating or deserted coal mines by coal geologists, in a traditional firepit of an old house. Indoor air particulate samples will be collected for nanoquartz and polycyclic aromatic hydrocarbons (PAHs) analyses. Bulk quartz content will be quantified by X-ray diffraction analysis. Size distribution of quartz will be examined by electron microscopes and by centrifugation techniques. Lifetime cumulative exposure to nanoquartz will be estimated for each subject. Using the epidemiology data, we will examine whether the use of C1 coal and the cumulative exposure to nanoquartz are associated with an elevated risk of lung cancer.

**Discussion:**

The high incidence rate of lung cancer in Xuan Wei, one of the counties in the current study area, was once attributed to high indoor air concentrations of PAHs. The research results have been cited for qualitative and quantitative cancer risk assessment of PAHs by the World Health Organization and other agencies. If nanoquartz is found to be the main underlying cause of the lung cancer epidemic in the study area, cancer potency estimates for PAHs by the international agencies based on the lung cancer data in this study setting should then be updated.

## Background

**Figure 1:**
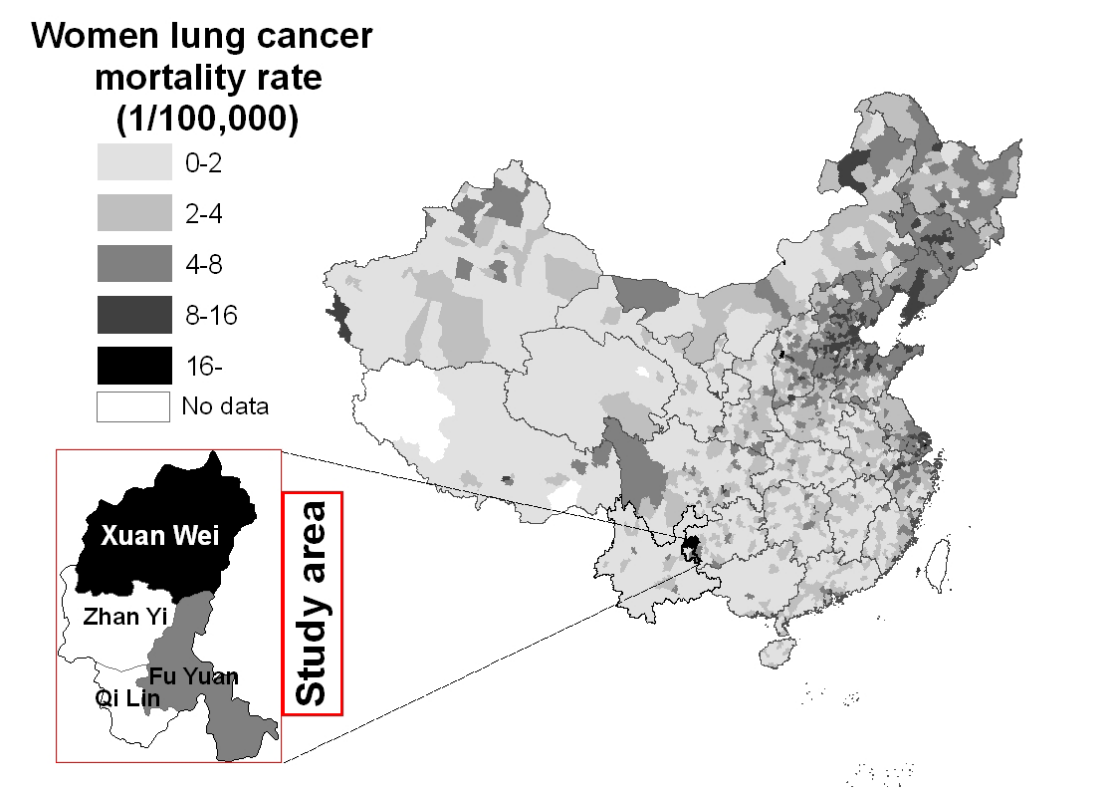
Map of China, showing county-specific annual female lung cancer mortality rates in 1973–75; the study area includes four counties as highlighted.

Lung cancer is the leading cause of cancer mortality worldwide [1]. China has a rapidly rising rate of lung cancer that will drive up global rates of lung cancer in the future [1]. Lung cancer mortality is generally higher in cities than in rural areas, but the highest female lung cancer rate of China was found in a rural county, Xuan Wei County of Yunnan Province (Figure 1). The mean age of lung cancer diagnosis was about 55 years, more than 10 years younger than in other areas, and the sex ratio of lung cancer mortality rates between males and females was 1.09, compared to the national average of 2.09 [2]. The abnormally high female lung cancer rate in this area is particularly noteworthy because there are few industrial exposures in this rural area, essentially all women are non-smokers, cigarette use has been associated with only a relatively low relative risk in several studies [3-6].

A series of etiology studies were carried out by investigators from the Chinese Academy of Preventive Medicine (CAPM) in cooperation with the United States Environmental Protection Agency (USEPA) [7]. The use of bituminous coal ("smoky coal" in early literature), compared to anthracite ("smokeless coal") and wood, was found to be a risk factor of lung cancer in Xuan Wei. In a survey of past fuel use and lung cancer mortality rates in 11 townships, lung cancer mortality rate was found to be correlated with the percentage of homes in the commune using bituminous coal [3]. A case-control study carried out in Xuan Wei in the 1980's showed an association between bituminous coal use and lung cancer [3]. A retrospective cohort study of 40,000 subjects carried out in 1992 further confirmed the link between bituminous coal use and lung cancer and showed that installing a chimney that vented smoke to the outside was associated with a reduction in lung cancer risk [6].

The polycyclic aromatic hydrocarbons (PAHs) concentrations in the air were the highest in bituminous coal smoke, followed by wood smoke and anthracite smoke [7]. The PAH fraction from a bituminous coal sample accounts for 43% of the organic mass and 61% of the total mutagenicity [8]. However, no correlation was found between contents of benzo(a)pyrene (BaP), the index compound of PAHs, and tumorigenic activity [9]. PAH-DNA adduct formation in placenta was not higher in women using bituminous coals than those using wood, and no dose-response relations between BaP exposure and placental DNA adduct levels or percentage of samples with detectable DNA adducts were found [10]. Other concomitant carcinogens besides PAHs in the bituminous coal emissions may also play an important role in the female lung cancer epidemic in Xuan Wei.

A controlled laboratory system was developed to burn fifteen types of coal in a manner similar to the way these fuels were used in Xuan Wei, and the PAHs, free radicals, transition metals, and crystalline silica in soot emissions were measured [11,12]. In an ecological epidemiology study, no association was found between the lung cancer risk and the exposures to PAHs, free radicals, or transition metals. It was the exposure to crystalline silica embedded in soot particles that was linked to lung cancer [11].

**Figure 2:**
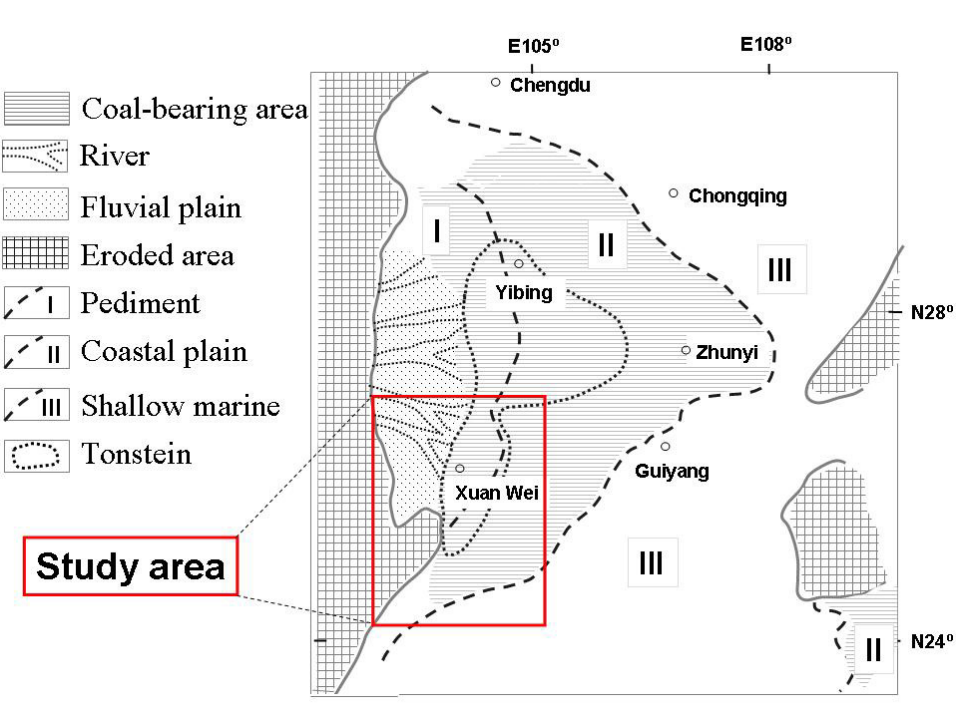
The sedimentary paleogeography of the coal-bearing deposits of Late Permian Changxing Formation in southwest China.

**Figure 3:**
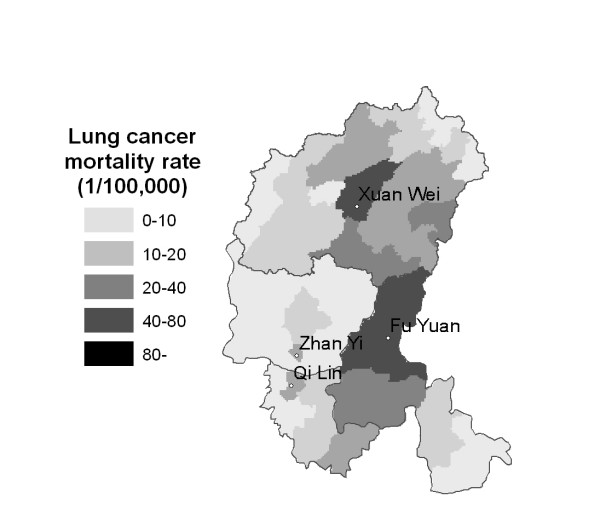
Map of the study area, showing the unadjusted lung cancer incidence rates (both genders) of the 57 townships in 2002.

**Figure 4:**
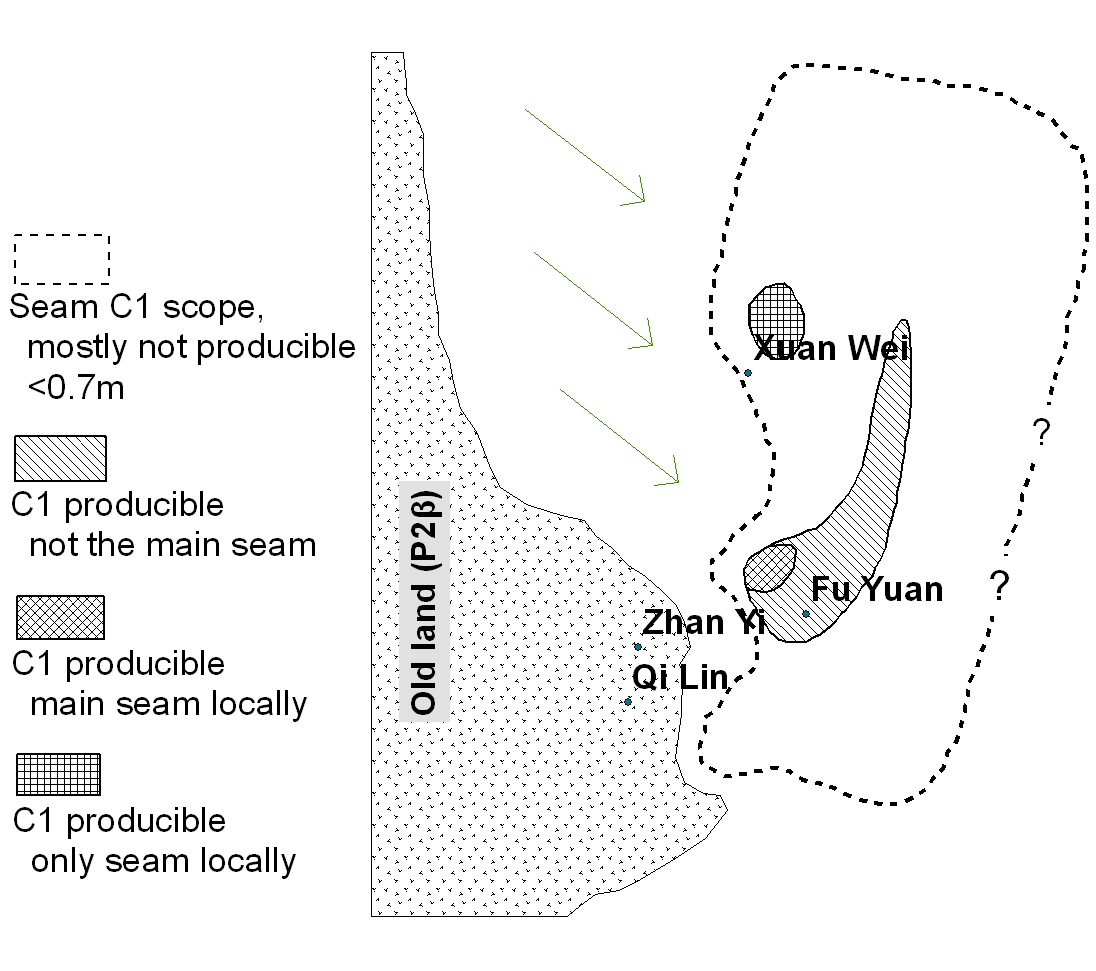
The sedimentary paleogeography of the C1 coal, the uppermost coal seam of Late Permian in southwest China.

**Figure 5:**
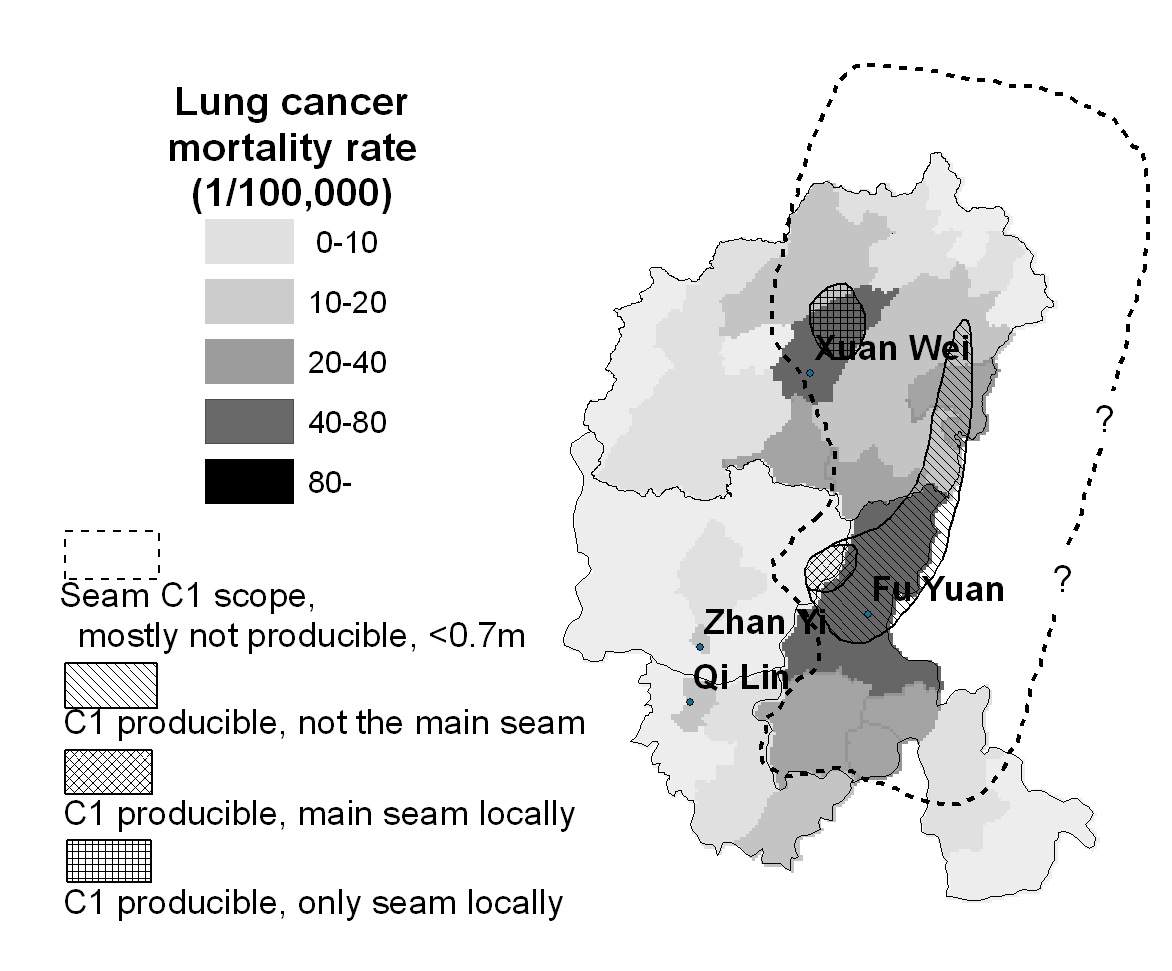
**An overlay of the data for lung cancer mortality rates (Fig. 3) and C1 coal distribution (Fig. 4)**. Lung cancer rates tend to be higher in places where C1 coal is produced.

The research area of Xuan Wei County has been expanded to the Pearl River Origin area that consists of 4 counties (Xuan Wei, Fu Yuan, Zhan Yi, and Qi Lin) (see Figure 1). The study power can be improved by increasing the variability of both lung cancer risk and exposure levels. The research team has collected some preliminary township-based lung cancer data and coal geology data (Figure 2) in the study area. The lung cancer map (Figure 3) was overlaid with the Late Permian C1 coal map (Figure 4) to construct Figure 5. Lung cancer rates tend to be higher in places where C1 coal is produced. Therefore, the research team has hypothesized the two processes: C1 coal combustion → crystalline silica in ambient air → lung cancer excess in non-smoking women.

**Figure 6:**
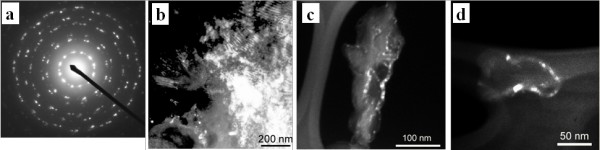
**SAED pattern and DF images of quartz in C1 coal**. (a) Typical SAED pattern (d = 1 μm) of the multiple quartz crystals in C1 coal; (b) DF image of quartz in parabolic or parallel fiber bundles; (c) and (d) DF images of quartz showing each fiber has a diameter of about 5 nm and is composed of crystallites 2*5 nm in size.

**Figure 7:**
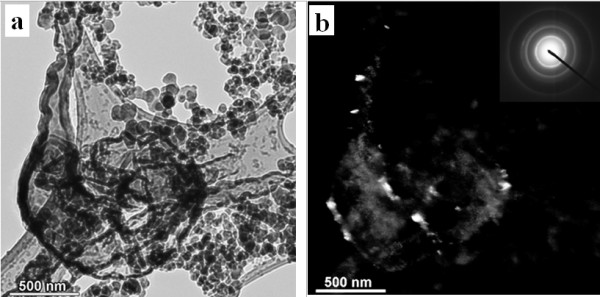
**Bright-field (a) and dark-field (b) images of quartz embedded in soot emissions from coal combustion**. Insertion in (b) shows the SAED pattern of quartz from an area of 1 μm.

**Figure 8:**
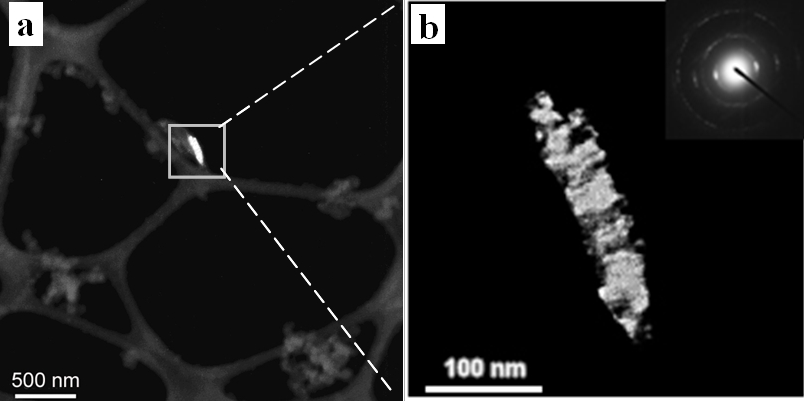
**Dark-field images of quartz embedded in soot emissions from coal combustion**. Highlighted area in (a) is expanded to (b). Insertion in (b) shows the SAED pattern of quartz from an area of 1 μm.

**Figure 9:**
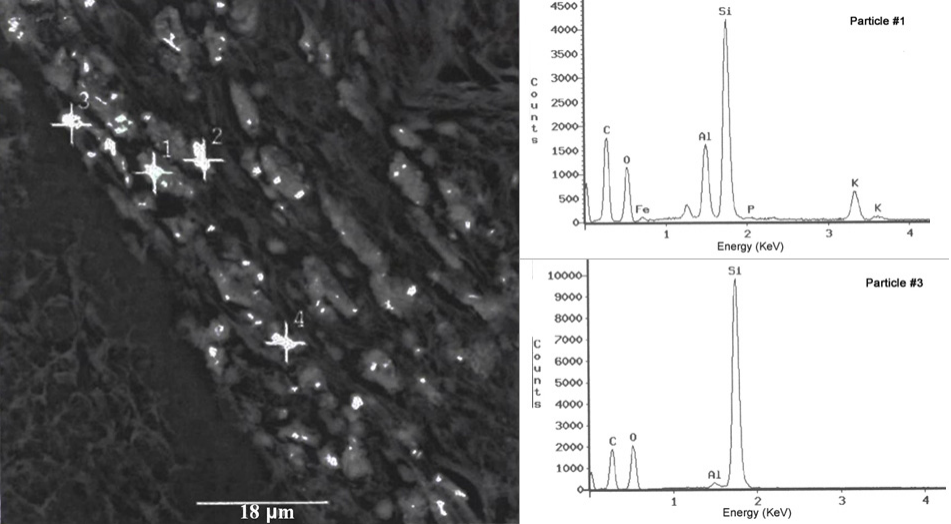
SEM image showing the dust deposited in the lung cancer tissue of one 56-year old housewife who used C1 coal for cooking and heating, and the EDX spectra of the numbered spots (1 and 3). The #1 and #3 particles contain free silica.

Quartz in C1 coal has been found to be microfibrous; each fiber has a diameter of about 5 nm and is composed of crystallites 2*5 nm in size (Figure 6). Nano-sized quartz has also been identified in soot emissions from C1 coal combustion (Figures 7 &8). Some alpha-quartz crystals may have been transformed to beta-quartz during combustion (Figure 7a). The small size of the quartz crystals allows them to be carried into air and inhaled by the women. The lung adenocarcinoma sample of a 56-year old housewife who used C1 coal for cooking and heating was examined under light microscope (LM); abundant birefringent dust including much crystalline silica was observed in the tissue [11]. The presence of silica particles in the lung tissue was also confirmed by Scanning Electron Microscopy coupled with Energy Dispersive X-ray analyzer (SEM-EDX) (Figure 9).

The tentative hypothesis generated in the previous ecological study has not been tested in an analytical epidemiology study. The actual indoor air quartz concentrations in a home where C1 coal and other types of coal are burned have not been measured. No data are available for the exact size distribution of quartz in coal combustion products. In fact, it is unknown whether the occurrence of nanoquartz is unique to the C1 coal; the current belief is that nanoquartz is also present in other types of coal, yet in a smaller amount. The detailed exposure assessment information should be integrated into the analytical epidemiology study to obtain further insights into the relationship between nanoquartz and lung cancer.

## Methods/Design

The research hypothesis involves two processes: household use of Late Permian C1 coal → nanoquartz in ambient air → lung cancer of non-smoking women. This research project consists of three components: an epidemiology study to examine the association between C1 coal use and lung cancer, an exposure assessment of nanoquartz in ambient air, and a dose-response assessment of the relationship between nanoquartz and lung cancer by integrating the epidemiology and exposure assessment data.

### I. C1 coal and lung cancer – a retrospective cohort study

#### Study setting

A retrospective cohort epidemiology study will be conducted in the Qujing District of Yunnan Province which is the origin of the Pearl River. There are a total of 9 counties in Qujing District, four counties of which will be chosen as the study area (Xuan Wei, Fu Yuan, Zhan Yi, and Qi Lin) (Figures 1 and 3). Among them, Xuan Wei County has the highest female lung cancer mortality rate and Fu Yuan County has one of the highest female lung cancer rates in China (Figure 1). Life-long residents of these four counties are considered the base population. The study area, including 764 administrative villages, had a population of 3.1 million in 2000.

The risk factor of interest is the use of C1 coal, which is producible in only a few places in the study area (Figure 4). In most cases, families obtain coal from within a few kilometers of their homes and stick to the use of one particular type of coal for at least a few years. In those places where C1 coal is producible, production varies with time depending on the availability of alternative types of coal or wood. Historical records from county-level administrations of coal exploration and local coal companies will be retrieved to compile a detailed inventory of the coal mines in operation during 1930–2009 in the study area. The cumulative exposure to C1 coal will be estimated for each subject by integrating information from the coal production inventory and the subjects' dwelling and coal use history.

#### Sample size

Sample size is calculated based on the primary hypothesis of the link between C1 coal use and lung cancer risk. The prevalence of C1 coal users in the study area was estimated to be approximately 20% (see Figure 4). According to the preliminary data shown in Figure 5, the incidence rate of lung cancer among the women who did not use C1 coal in the whole study area was estimated to be 10/100,000. For a statistical power of 0.8, alpha = 0.05, and using chi-square test, the required person years of follow-up would depend on the expected relative risk (RR) for the use of C1 coal (Table 1):

**Table 1:** Relative risk (RR) for the use of C1 coal

Expected RR	Required person-years
1.5	466,000
2.0	134,000
3.0	42,000
5.0	15,000

The use of C1 coal is expected to be a relatively strong risk factor for lung cancer. In a previous ecological study, the lung cancer mortality rate was 244.6/100,000 among women using C1 coal and 43.9/100,000 among women using other types of coal in Xuan Wei [11]. Assuming there is no cross-level fallacy in this setting, we would expect the relative risk (RR) of lung cancer for the use of C1 coal to be around 5. A simple random sample of 18,000 person-years would be sufficient to detect the effect of C1 coal on lung cancer development.

However, cluster sampling will be used in the current study in stead of simple random sampling. Subjects in the same household or village tend to be similar to one another. The sample would not be as varied as it would be in a simple random sample. To compensate for the loss of effectiveness of cluster sampling (design effect), we need to increase the calculated sample size by at least threefold. Furthermore, the sample size should be sufficient to allow for the adjustment of potential confounding factors such as passive smoking and dietary habits. For a weak risk factor with a RR of 2.0, the required sample size would be 134,000. The sample size should be increased further to reflect such factors as loss to follow-up, response rate, and lack of compliance etc. Taken together, the required sample size would be approximately 150,000 person-years for the cohort study. To reach this sample size, a cohort of 12,000 subjects will be followed up for 14 years, from 1995 through 2009.

#### Study cohort

The study population will include those female subjects born during 1930–1965, living in the study area since January 1, 1995. The computerized records in the local hospitals were not set up until 1995. The sampling frame will be the 764 villages stratified by county (Xuan Wei, Fu Yuan, Qi Lin, and Zhan Yi) and the availability of producible C1 coal. A stratified cluster sample will be taken to construct the study cohort. One village has approximately 500 women eligible for the study; a random sample of 24 villages will be selected from the study area.

The cohort will be followed up until January 1, 2009. Deaths and emigrations will be identified by linkage with the local administrative records, and incident lung cancer cases by linkage with hospital records. There is not a cancer register in the study area; records of the local hospitals will be searched to identify all diagnoses of lung cancer among the study subjects. All diagnoses will be reviewed by a panel of radiologists and pathologists. Each person contributes to the person years at risk from January 1, 1995 until the date of diagnosis of lung cancer, death, emigration, or January 1, 2009, whichever comes first.

The questionnaire will ascertain life history of household fuel use (coal type and coal mine name), stove type (unvented firepits or stoves with chimney), cooking practice (age of starting to cook, years of cooking), smoking (active and passive), education, residence locations, time spent indoors and outdoors, dietary habits, and illnesses in subjects and their relatives. Fifty interviewers (two per village) who were involved in the national mortality survey in 2005 will be trained to administer a structured questionnaire for all the study subjects in the 24 villages. Each subject will be interviewed in person when feasible; when the subject is deceased or severely ill, surrogate respondents will be interviewed. Informed consent will be obtained from all the respondents. Ethical approvals will be obtained from the Institutional Review Boards of Yunnan Province Tumor Hospital and the Chinese University of Hong Kong.

#### Statistical analysis

Cox regression models will be constructed (SAS PHREG procedure) to estimate the Relative Risks (RR) with 95% confidence intervals (CI). The outcome event will be the diagnosis of lung cancer (incident lung cancer), and the main predictor of interest will be C1 coal use. Duration of exposure and cumulative exposures to C1 coal and nanoquartz will be treated as a time-dependent variable in Cox regression analysis. Potential confounding factors will be included in the model: 1) age; 2) education; 3) active and passive smoking; 4) dietary habits; 5) cumulative exposures to PAHs from coal combustion; and 6) family lung cancer history.

### II. Nanoquartz emissions from C1 and other coals – exposure assessment

#### Coal sampling

We will list all the coal seams ever used by the study subjects according to the questionnaires (life history of fuel use: coal type, coal mine name and residence locations) of the epidemiology study and historical records of the coal companies. For each coal seam, sample will be obtained by the coal geologists, from the currently operating or deserted coal mines. There are more than 30 Late Permian coal seams, including the C1 coal seam of particular interest, in the study area; we estimate that there have been mainly 10 seams being mined and used by the local residents in the past decades. There are also some isolated coal deposits from the Carboniferous Period. A single seam may be accessed through multiple mines, and one coal mine may access different coal seams at different times. We expect to obtain approximately 50 coal samples from the 24 villages included in the epidemiology study. Coal samples will be subjected to proximate, ultimate, and ash analyses.

#### Coal combustion by firepit and indoor air sampling

Given the long latency period for lung cancer development, it is the historical exposure to potential carcinogens that can be related to lung cancer. Prior to 1973, 99.9% of the households in Xuan Wei used unvented firepits [2]. In recent decades, most of the households in Xuan Wei use vented stoves [6]. Measurement of the current exposure of the non-smoking women in the study area cannot represent their historical exposure to relevant carcinogens because the type of stove (unvented and vented) and coal (from different seams mined over the years) are not the same as before. Coal combustion experiments will be conducted to simulate the subjects' historical exposure.

An unvented firepit in an old house in the study area will be selected to conduct the coal combustion experiments to simulate the cooking practice. One experimental cycle will involve heating 3 kg of water from room temperature to its boiling point and then simmer for 20 minutes. Three experiments will be conducted each day to simulate the three meals. For each coal sample, the experiments will be repeated for three days. Indoor air particulate matter with aerodynamic diameter less than 2.5 μm (PM_2.5_) will be collected for further chemical and physical analysis. Emission factor and indoor air concentration of PM_2.5 _will be measured for each coal sample.

Large amounts of PM_2.5 _are necessary to extract quartz for quantitative analysis by XRD. The detection limit for quartz by NIOSH Method 7500 [13] and OSHA ID142 [14] is 5 μg. Ambient aerosols from the EPA Inhalable Particle Network (IPN) contained 0.4 (± 0.7) weight percent as quartz in the PM_2.5 _fraction [15]. Quartz content in the soot emissions from coal combustion ranged from 0.01 to 0.09 weight percent [11]. A minimum of 50 mg PM_2.5 _per sample would be required for quartz quantification.

A PM_2.5 _sampler with a flow rate of 100 L/min will be used to collect PM_2.5 _on pre-weighted 5.0 μm polyvinyl chloride (PVC) filters (88 mm in diameter). With a typical ambient PM_2.5 _concentration of 2 mg/m^3 ^during firepit combustion [2,11], a sampling period of 250 minutes would be necessary to collect 50 mg of PM_2.5_. One 8-hour sample will be collected per day; PM_2.5 _collected on serial filters in one day will be pooled for analysis. PM_2.5 _collected on PVC filters will be used for XRD analysis of quartz. Another identical PM_2.5 _sampler will be used to collect PM_2.5 _on glass fiber filters for PAH analysis.

#### Coal combustion in tubular furnace and ash collection

Indoor air PM_2.5 _samples reflect the real life exposure of the study subjects to coal smoke; the mass of an indoor air PM_2.5 _sample (≥50 mg) will suffice the need for bulk analysis of quartz content. The amount, however, will not be sufficient if we want to use centrifugation techniques to separate the PM_2.5 _into nano-sized and coarser fractions and subsequently measure the nanoquartz fraction in PM_2.5 _by XRD.

As a remedial strategy to obtain large amounts of nanoquartz with less interference from carbonaceous materials (e.g., in coal smoke), we will burn approximately 10 g of coal in a horizontal tubular furnace and collect the remaining coal ash for size fractionation and quartz analysis. Coal powder samples (100~200 μm in particle size) will be evenly put into a high temperature-resistant (1300°C) transparent quartz tube and heated to 550°C by high-temperature furnace under the conditions of mixed gas ventilation (the ratio of N_2 _to O_2 _is 4:1) until the coal residue keeps a constant mass.

#### Analysis of quartz in PM_2.5 _and coal ash

Quartz analysis will be performed by X-Ray Diffraction (XRD) following modified procedures of NIOSH 7500 and OSHA ID-142 methods [13,14].

PM_2.5 _samples collected on PVC filters will be placed in a centrifuge tube, the filter and any organic materials will be dissolved with tetrahydrofuran (THF), and the solids will be suspended in the THF using an ultrasonic bath. The solids will be transferred onto silver filters for XRD analysis. Some alpha-quartz crystals may have been transformed to beta-quartz during combustion; preliminary data show the fraction is not significant. NIST SRM 2950 calibration set (alpha-quartz) will be used for preparing working standards at known concentrations. The inorganic solids extracted from PM_2.5 _will also be deposited on TEM grids as a single layer. Size distribution of quartz will be estimated by analyzing one hundred quartz particles in consecutive random fields with SEM-EDX [16]. The particle diameter will be estimated by SEM, and elemental composition determined by EDX. The crystallinity and morphology of silica particles will be analyzed with TEM.

Coal ash samples will be subjected to centrifugation in order to separate nano-sized particles from coarser ones [17,18]. Size distributions will be examined by SEM and TEM. Fractions of different sizes will be analyzed by XRD for quartz quantification.

#### Analysis of PAHs in PM_2.5_

PM_2.5 _on glass fiber filters will be analyzed by Gas Chromatography/Mass Spectrometry (GC/MS) to determine the concentration of benzo [a]pyrene (BaP), the index compound of carcinogenic PAHs. Concomitant exposure to PAHs will be adjusted for when analyzing the relationship between nanoquartz exposure and lung cancer risk.

### III. Nanoquartz and lung cancer – risk assessment

#### Dose-response relationship between nanoquartz and lung cancer

Combining the coal use history and indoor air concentrations of nanoquartz associated with different coal types, we will calculate one subject's cumulative exposure to nanoquartz. The same calculation will be performed for concomitant PAH carcinogens. Cox regression models will be developed for relating the cumulative exposures to the lung cancer data. Potential confounding factors such as passive smoking, dietary habits, and exposure to PAHs will be included in the model. Cumulative exposure to nanoquartz will be treated as a continuous variable in the model to test whether a dose-response relationship exists between nanoquartz exposure and lung cancer risk. Cox regression models will be constructed (SAS PHREG procedure) to estimate RR of lung cancer for a unit increase of nanoquartz exposure.

#### Analysis of nanoquartz in lung tumors by electron microscopy

Identification of nanoquartz in lung tumors would lend plausibility to the hypothesis of nanoquartz causing lung cancer in the study area. Tumor tissue samples of the lung cancer cases in the cohort will be collected for quartz deposition examination under electron microscopes. Currently we have collected 10 lung tumor samples from the study area to set up and test the microscopy analysis procedures. Lung cancer tissue blocks of roughly 1 mm on one side will be fixed in glutaraldehyde in cacodylate buffer, postfixed in osmium tetroxide, dehydrated with alcohol, embedded in Epon, cut into thin sections (0.1 μm and 0.5 μm, respectively), and mounted on copper grids [19]. The thin section 0.5 μm will be used for analysis in the mode of Scanning Transmission Electron Microscopy (STEM); the ultrathin section (100 nm) will be used for routine TEM analysis.

The TEM grid samples will be firstly examined by a scanning electron microscope (SEM) equipped with an energy-dispersive X-ray detector (EDX). Mineral identification will be inferred from morphology and elemental composition of the grains. Then the TEM grids will be examined in an analytical TEM operated at 200 keV accelerating potential. The composition of particles in crystalline phases will be determined by the d-spacing measurements from the SAED patterns. For a complex aggregate, dark-field imaging will be applied to examine the separate phases using specific reflection rings or spots in the Selected Area Electron Diffraction (SAED) pattern [20]. Silica crystals are highly sensitive to electron irradiation damage. Under the high-energy electron-beam, the microcrystalline SiO_2 _samples showed visible damage within seconds to minutes before becoming amorphous [21]. To minimize irradiation damage, relatively low magnifications will be chosen to examine the crystals. Charge-coupled device (CCD) camera will be used to record images.

## Discussion

The objectives of this project include: 1) to examine whether the domestic use of Late Permian C1 coal is associated with the high lung cancer incidence of non-smoking women in the Pearl River Origin area; 2) to examine whether indoor air concentrations of nanoquartz is higher when C1 coal is burned than when other types of coal are burned indoors; and 3) to examine whether nanoquartz exposure is associated with increased lung cancer risk. The study hypothesis involves two processes: C1 coal combustion → nanoquartz in ambient air → lung cancer excess in non-smoking women.

Confirmation of the hypothesis above will have significant long-term local and global impacts. In the study area, the C1 coal should be banned for residential use. Stove improvement by installing a chimney and venting the coal smoke outdoors has been effective in reducing indoor air pollution levels and reducing the lung cancer risk in some townships within the study area. The lung cancer mortality rates in a larger area, however, have been on a rise in recent decades. One underlying cause could be that the low quality C1 coal starts to be mined in a larger area and used by more local households. For practical reasons, the C1 coal can be banned for residential use; it can be used exclusively for certain industries. For example, the submicron size of quartz and their intimate mix with organic matrix in the coal are favorable conditions for silicon carbide production.

Worldwide, findings from this study would lend more support to the link between crystalline silica exposure and lung cancer risk. In 1996, there was not a unanimous agreement among the International Agency for Research on Cancer (IARC) Working Group on the classification of crystalline silica, inhaled in the form of quartz or cristobalite from occupational sources, as a Group 1 human carcinogen. The variability of quartz hazard may be related to source, size, morphology, surface chemistry, and biopersistance. The current study is expected to provide new evidence and insights for the carcinogenicity of quartz. Moreover, the silica hazard in non-occupational settings will obtain more attention by the international community. Nanoquartz embedded in the soot particles in this study setting may be a special case; whether this form of quartz occur in other ambient air settings should be investigated in order to protect the general population.

Another perspective of the current study is on the carcinogenicity evaluation of polycyclic aromatic hydrocarbons (PAHs). The high mortality rate of lung cancer in Xuan Wei, one of the counties in the current study area, was once attributed to high indoor air concentrations of PAHs. The research results have been widely cited for qualitative and quantitative cancer risk assessment of PAHs by the World Health Organization and other agencies. However, the link between PAHs and lung cancer in Xuan Wei was not reproduced in a later ecological correlation study and will be examined again in the current analytical epidemiology study. If nanoquartz is found to be the main underlying cause of the lung cancer epidemic in the study area, cancer potency estimates for PAHs by the international agencies based on the lung cancer data in this study setting should then be updated. Beyond the scope of the current study, experimental research will be needed to examine the potential interaction between these two concomitant carcinogens in soot emissions: quartz and PAHs.

Nanoquartz has been synthesized in hydrothermal conditions to study whether a well known toxic particle such as quartz would demonstrate a higher toxicity as a nanoparticle. Unexpectedly, the synthesized nanoquartz was found to be less toxic than micron-sized quartz, even when the effects were compared on a unit weight base. The source of the crystal affects the variability of quartz hazard. The naturally occurring nanoquartz extracted from the C1 coal and emission samples in the current study may serve as an important set of reference materials for experimental studies of nanoparticle toxicity.

## Competing interests

The authors declare that they have no competing interests.

## Authors' contributions

LT conceived of the study, collected the preliminary data and drafted the manuscript. SD and JW participated in the study design and helped to draft the manuscript. YH and SCH made contributions to the study design and preliminary data collection. YZ, DL and CPK participated in the preliminary data collection. All authors contributed to the writing of the manuscript and approved the submitted version of the manuscript.

## Pre-publication history

The pre-publication history for this paper can be accessed here:


